# High-Yield Production of the Major Birch Pollen Allergen Bet v 1 With Allergen Immunogenicity in *Nicotiana benthamiana*

**DOI:** 10.3389/fpls.2020.00344

**Published:** 2020-04-02

**Authors:** Yuki Yamada, Masanori Kidoguchi, Akira Yata, Takako Nakamura, Hideki Yoshida, Yukinori Kato, Hironori Masuko, Nobuyuki Hizawa, Shigeharu Fujieda, Emiko Noguchi, Kenji Miura

**Affiliations:** ^1^Graduate School of Life and Environmental Sciences, University of Tsukuba, Tsukuba, Japan; ^2^Department of Medical Genetics, Faculty of Medicine, University of Tsukuba, Tsukuba, Japan; ^3^Division of Otorhinolaryngology and Head and Neck Surgery, Department of Sensory and Locomotor Medicine, Faculty of Medical Science, University of Fukui, Fukui, Japan; ^4^Tsukuba-Plant Innovation Research Center, University of Tsukuba, Tsukuba, Japan; ^5^Department of Respiratory Medicine, Faculty of Medicine, University of Tsukuba, Tsukuba, Japan

**Keywords:** allergy, agroinfiltration, birch, plant-based protein expression system, Tsukuba system

## Abstract

Type I allergy is an immunological disorder triggered by allergens and causes significant health problems. The major allergen of birch pollen is Bet v 1, which belongs to the pathogen-related protein 10 (PR-10) family. Here, we established a rapid and robust method for the production of Bet v 1 in *Nicotiana benthamiana* leaves, with binding activity to allergic patients’ IgE. The Bet v 1 allergen was expressed in *N. benthamiana* using a strong agroinfiltration-based transient protein expression system, which consists of a deconstructed geminiviral vector system with a double terminator. Five days post-infiltration, the allergen concentration in *N. benthamiana* leaves was 1.2 mg/g of fresh mass, being this the maximum yield of Bet v 1 in plants reported up to now. A part of plant-derived Bet v 1 was glycosylated. Bet v 1 purified from *N. benthamiana* or *Brevibacillus brevis* was used to carry out enzyme-linked immunoassays; both recombinant allergens were found to have comparable binding properties to the IgE of allergic patients. These results suggest that our plant expression system allows rapid and robust production of the allergen, which keeps the immunogenicity.

## Introduction

Type I allergy or immediate hypersensitivity is the consequence of the interaction between an allergen and immunoglobulin E (IgE) antibodies, which are attached to mast cells. Allergic symptoms, including allergic asthma, rhino-conjunctivitis, and atopic dermatitis, significantly affect human health and a quality of life. It is estimated that 300–400 million people in the world is affected by type 1 allergies, causing 120 billion Euros in health care costs, sick leaves, and economic losses (Calderon et al., 2012). These symptoms are elicited by environmental aeroallergens such as pollen or house dust mites. Birch pollen is widespread in Europe, North America, Russia, and northern Japan. More than 100 million patients suffer from allergy to birch pollen (Moverare et al., 2005). The major birch pollen allergen is Bet v 1.

The Bet v 1 allergen is a member of the plant pathogenesis-related proteins family PR-10 (Kleine-Tebbe et al., 2017). Pathogenesis-related proteins are induced by different plant stresses, including pathogen attack (Kim et al., 2008), and may be important for plant defense. It was reported that overexpression of rice PR-10 promoted decreased rice susceptibility to blast fungus (Wu et al., 2016). Bet v 1 homologs are included among various plant-derived food allergens such as apple Mal d 1, hazelnut Cor a 1, cherry Pru av 1, soybean Gly m 4, and tomato Sola I 4 (Kleine-Tebbe et al., 2017). The patients having birch pollen allergy usually suffer from secondary pollen food syndrome, which negatively impacts on their health-related quality of life (Popescu, 2015). For example in Germany, the mean of “Food Allergy Quality of Life Questionnaire – Adult Form” (scale from 1 to 7, no impairment – maximum impairment) score was 3.7, indicating mild to moderate impairment of health-related quality of life (Beyer et al., 2016).

Allergen immunotherapy is based on the repeated administration of disease-eliciting allergens to modulate the allergic immune response (Akdis and Akdis, 2014). During AIT, allergen-specific immunoglobulin G, particularly IgG_4_, is induced (Wachholz and Durham, 2004). IgG_4_ competes with IgE to bind the allergen, leading to the inhibition of the IgE-mediated hypersensitive mechanisms to alleviate allergic symptoms (Mobs et al., 2012; Shamji et al., 2012). Bet v 1A-specific IgG_4_ developed by AIT competes with IgE for partly identical or largely overlapping epitopes (Groh et al., 2017). For AIT, recombinant forms of Bet v 1 were used, and they were found to be so efficient and safe as birch pollen extracts (Pauli et al., 2008; Nony et al., 2015). Those reports suggest that recombinant Bet v 1 protein may serve as a clinical allergen for therapeutic purposes.

Not only Bet v 1, many allergens are of plant origin. Thus, protein expression systems able to generate high levels of recombinant protein are required to produce plant allergens for therapeutic uses, given the need for downstream processing. A production level of 0.2 mg/g of fresh mass (FM) was previously reported in *Nicotiana benthamiana* leaves using the tobacco mosaic virus with a recombinant viral RNA containing the *Bet v 1* coding sequence (Krebitz et al., 2000). *Chlamydomonas reinhardtii*, a green alga, is one of the platforms to produce recombinant proteins. The yield of Bet v 1 in this platform was approximately 125 ng (0.125%) per 100 μg TSP (Hirschl et al., 2017). It is difficult to convert from% TSP to mg/g FM. Production of GFP at 4.0 mg/gFM in *N. benthamiana* is equivalent to 36% TSP (Yamamoto et al., 2018). That means yield of Bet v 1 at 0.2 mg/gFM in *N. benthamiana* (Krebitz et al., 2000) is approximately 1.8% TSP. Yield of Bet v 1 in *C. reinhardtii* was very low. If the protein is very precious and has high drug price, only small amount of protein is enough. But large amount of allergen (5–20 μg/treatment) is required for immunotherapy, because the optimum duration for allergy immunotherapy is around 3–5 years according to the document from World Allergy Organization. Transgenic rice expressing in the endosperm the Bet v 1 or the tree pollen chimera 7 (TPC7), a hypoallergenic derivative of Bet v 1, was generated by DNA shuffling (Wallner et al., 2007) and accumulated to a maximum protein level of 170 and 550 μg/seed, respectively (Ogo et al., 2014). Seventy seeds are about 2 g., thus, the yields of Bet v 1 were approximately 6 and 19 mg/g seed, respectively. Rice endosperm is a good platform to produce recombinant proteins. But is should be considered that production of 2 g rice seeds is required for about 150 g of shoot FM of rice (Takehisa et al., 2004), thus, the calculation should be 0.08 and 0.26 mg/g total FM in rice. These data suggest that the production of Bet v 1 in *N. benthamiana* and in cereal crops are competitive. On the other hand, this technology requires land use and time to obtain enough rice seeds. The cultivation period of *N. benthamiana* is approximately 4 to 6 weeks, which is much shorter than that of rice, typically 20 weeks. When recombinant proteins are produced in leaves, a considerable amount of biomass can be obtained from a single plant compared to seed-based systems. It takes at least 4 months to make transgenic rice, whereas, it takes 3 to 14 days to obtain the protein of interest after agroinfiltration to *N. benthamiana*. And the several high expression systems in *N. benthamiana*, such as magnICON technology and Geneware viral vector technology, have been established (Gleba et al., 2014). Because of these reasons, *N. benthamiana* transient expression systems are often commercially used to obtain recombinant proteins (Gleba et al., 2014).

We previously developed one of the most efficient transient protein expression systems in plant cells, the ‘Tsukuba system’ (Yamamoto et al., 2018; Hoshikawa et al., 2019; Suzaki et al., 2019). By using this system, approximately 4 mg of GFP per g FM was produced in *N. benthamiana* leaves. The vector for the Tsukuba system is pBYR2HS, which contains a geminiviral replication system and a double terminator (Yamamoto et al., 2018). This system is applicable to different plant species and varieties, including eggplants, peppers, lettuces, melons, orchids (Yamamoto et al., 2018), wild tomatoes (Hoshikawa et al., 2019), and leguminous plants (Suzaki et al., 2019); however, massive expression levels of target proteins were achieved in *N. benthamiana* among several kinds of plants. The peak of protein accumulation, about 3 days after agroinfiltration, by using the Tsukuba system (Yamamoto et al., 2018) is earlier than that, about 5 days after agroinfiltration, by using the magnICON system, a tobamovirus (TMV)-based deconstructed viral system engineered to achieve high levels of recombinant protein in *N. benthamiana* (Marillonnet et al., 2005).

In this study, we produced recombinant Bet v 1 protein in *N. benthamiana* and the bacterial host *Brevibacillus brevis*, and compare their allergen yields, as well as the immunogenicity of the recombinant proteins obtained when exposed to sera of human patients.

## Materials and Methods

### Preparation of the *B. brevis* Expression System

A natural or a *B. brevis* codon-optimized *Bet v 1A* gene (accession number of Bet v 1, P15494.2) was synthetically produced using the GeneArt Strings DNA Fragments service (Thermo Fisher Scientific) and amplified with the primers pBIC-HIS-Betv1wt-F and pBIC-HIS-Betv1wt-R or with pBIC-HIS-Betv1ABb-F and pBIC-HIS-Betv1ABb-R (Supplementary Table S1). The PCR product was inserted into pBIC1, pBIC2, pBIC3, and pBIC4 using *B. brevis* competent cells, according to the manufacturer’s instructions (*Brevibacillus* Expression System, Takara Bio). After confirming the gene sequence of the insertion, *B. brevis* was cultured in 10 mL of 2SYNm medium (20.0 g/L glucose, 40.0 g/L bacto soytone, 5.0 g/L bacto yeast extract, 0.15 g/L CaCl_2_⋅2H_2_O, and 50 mg/L neomycin) or in 10 mL of TMNm medium (10.0 g/L glucose, 10.0 g/L phytone peptone, 5.0 g/L Ehrlich bonito extract, 2.0 g/L yeast extract, 10 mg/L FeSO_4_⋅7H_2_O, 10 mg/L MnSO_4_⋅4H_2_O, 1 mg/L ZnSO_4_⋅7H_2_O, and 50 mg/L neomycin) at 33°C for 3 days with shaking at 180 rpm. For large-scale incubation, 500-mL of liquid medium was prepared in 3-L of Erlenmeyer flask and *B. brevis* was incubated at 33°C for 3 days with shaking at 160 rpm on a rotary shaker (TAITEC Bio-Shaker BR-300LF, Japan).

### Preparation of the *N. benthamiana* Expression System

A *Nicotiana tabacum* codon-optimized *Bet v 1A* gene fused with 6x His tag and DYKDDDDK tag was synthetically produced using the GeneArt Strings DNA Fragments service (Thermo Fisher Scientific) and amplified with the primers pBYR2HS-His24 and pRI201-Betv1ANt-R (Supplementary Table S1). The PCR product was inserted into the *Sal*I-digested pBYR2HS (Yamamoto et al., 2018) with an In-Fusion HD Cloning kit (Takara Bio). After confirmation of the sequence of the insertion, the pBYR2HS-HF-Betv1Nt plasmid was transformed into *Agrobacterium tumefaciens* GV3101. Pre-cultured *A. tumefaciens* GV3101 harboring the pBYR2HS-HF-Betv1Nt was transferred to L-broth media containing 10 mM MES (pH 5.6), 20 μM acetosyringone, 50 mg/L kanamycin, 30 mg/L gentamycin, 30 mg/L rifampicin and grown at 28°C overnight with shaking at 160 rpm on a rotary shaker (TAITEC Bio-Shaker BR-300LF, Japan) to the stationary phase. Subsequently *Agrobacterium* was resuspended in the infiltration buffer (10 mM MES (pH 5.6), 10 mM MaCl_2_, 100 μM acetosyringone) to adjust OD_600_ = approximately 0.5. A 500-mL portion of the *Agrobacterium* suspension was placed into a 500-mL glass beaker inside a vacuum desiccator. The leaves of 5-week-old *N. benthamiana* plants, grown in the cultivation room at 24°C under a 16-h light/8-h dark photoperiod, were immersed into the suspension and vacuum-infiltrated (29 inHg) for 5 min and then incubated for 3 to 7 days under the same conditions.

### Protein Purification

The *B. brevis* suspensions were centrifuged at 44,000 × *g* for 20 min at 4°C in a TOMY SRX-201 centrifuge using a TA-24BH rotor (Tokyo, Japan), and the supernatant filtrated with an Omnipore Membrane Filter (pore size 0.2 μm, Merck Millipore). After filtration, imidazole was added to a final concentration of 20 mM, and the pH was adjusted to 7.4. Four hundred mL of suspensions, which contains Bet v 1 fused with His tag, was purified by using the ÄKTA Start System equipped with a HisPrep FF 16/10 column (column volume: 20 mL, GE Healthcare).

The leaves of *N. benthamiana* agroinfiltrated with pBYR2HS-HF-Betv1Nt were frozen with liquid nitrogen and ground with mortar. A lysis buffer (50 mM Tris, pH 8.0, 100 mM NaCl, and 1 mM PMSF) was subsequently added in 1:5 proportion to resuspend the samples. These suspensions were incubated on ice with shaking for 1 h and filtrated with Miracloth (Calbiochem). After a centrifugation at 44,000 × *g* for 20 min at 4°C, ammonium sulfate was added to a final concentration of 30 (% saturation). This solution was incubated on ice with shaking for 1 h and centrifuged again. The supernatant was dialyzed with the dialysis membrane (molecular weight cut off: 10,000) to remove high concentration of ammonium sulfate and to replace with the binding buffer (10 mM Tris, pH 7.4, 0.5 M NaCl, 20 mM imidazole, and 1 mM PMSF). Bet v 1 fused with His tag was purified using the ÄKTA Start System equipped with a HisPrep FF 16/10 column (column volume: 20 mL, GE Healthcare).

After purification by HisPrep column, the purified protein in the peak fraction was concentrated and the buffer was changed to Tris-buffered saline (137 mM NaCl, 2.7 mM KCl, 24.8 mM Tris, pH 7.4) by using a 10 kDa cut-off membrane (Sartorius Vivaspin 20).

Bet v 1 from *N. benthamiana* was incubated in a reaction buffer with PNGase F or Endo H at 37°C for 1 h, according to the manufacturer’s instructions (New England Biolabs).

### SDS-PAGE and Immunoblot Analysis

Proteins were separated on SDS-PAGE. The gels were either stained with CBB or proteins were transferred onto a PVDF membrane (Amersham Hybond P PVDF, GE Healthcare). The blot was probed with anti-Bet v 1, mouse monoclonal 2E10 (Indoor Biotechnologies, Ltd.), or anti-6x His tag mouse monoclonal antibody (Thermo Fisher Scientific).

### ImmunoCAP Assay to Determine Specific Immunoglobulin E Antibody Levels

Serum samples obtained as control samples in our previous studies (Yatagai et al., 2013; Kanazawa et al., 2019) were collected and stored at −80°C until use. Among them, 14 serum samples whose specific IgE levels for birch allergen were available and specific IgE levels positive (≥class 1) were selected. Also, one sample with a specific IgE level for birch under the detection level (class 0) was tested. Therefore, a total of 15 serum samples were used to quantify Bet v 1-specific IgE levels using the ImmunoCAP assay (Thermo Fisher Diagnostics K.K., Tokyo, Japan) (Paganelli et al., 1998). This assay is the current standard method based on sandwich immunoassay to measure the specific IgE levels agains allergens and allergen components. Briefly, recombinant Bet v 1 covalently coupled to the solid phase reacted with the specific IgE in the patient serum sample, then non-specific IgE was removed in the washing step. After washing, β-galactosidase-labeled anti-IgE were added and incubated to form a complex. After washing to remove unbound enzyme-labeled anti-IgE, the bound complex was incubated with Development Solution. The reaction was terminated by adding Stop Solution, and the fluorescence of the eluate was measured. Human specific IgE values were calculated from IgE calibration curves. This study was approved by the research ethics committee of the University of Tsukuba and the University of Fukui.

### Recombinant Bet v 1-Specific IgE Quantification by ELISA

Recombinant Bet v1 derived from *B. brevis* or *N. benthamiana* was dissolved in phosphate-buffered saline (PBS) at 1 μg/ml. Then, 100 μl of recombinant Bet v1 solution was added to each well in a Nunc MaxiSorp flat-bottom 96-well plate (Thermo Fisher Scientific, Waltham, MA, United States). The plate was sealed and kept overnight at 4°C. Subsequently, the plate was washed with PBS containing 0.1% (v/v) Tween 20 (Wako Junyaku, Osaka, Japan; further designated as PBS-T), and the blocking buffer (PBS-T containing 1% (w/v) skim milk) was added to each well and incubated for 1.5 h at room temperature. After washing with PBS-T, 100 μl-aliquots of patients’ sera previously diluted to 20% (w/v) [using PBS-T containing 1% (w/v) skim milk] were added to the wells, followed by a further incubation of 1.5 h at room temperature. Then, the plate was washed with PBS-T containing 1% (w/v) skim milk and a 100 μl-aliquot of anti-human IgE-HRP conjugate (KPL, Gaithersburg, MD, United States) at a concentration of 0.1 μg/ml in PBS-T containing 1% (w/v) skim milk was added to each well. The plate was incubated for 1.5 h at room temperature and washed again with PBS-T, and the reaction was developed by adding 100 μl of 1-StepUltra TMB-ELISA (Thermo Fisher Scientific), with an incubation of 15 min at room temperature. The reaction was terminated by adding 100 μl of 2M H_2_SO_4_. Absorbance at 450 nm was measured by MPT–300 (Corona Electric, Co., Ltd., Ibaraki, Japan). The assay was carried out in triplicate.

### Statistical Analysis

At least three independent experiments were performed. The mean and variation, SEM, of protein production yields were calculated, based on the band intensity. Correlation between Bet v 1 IgE levels determined by ImmunoCAP and OD values in ELISA was evaluated by Spearman’s rank correlation analysis. If *P* < 0.01, we concluded that the correlation is statistically significant.

## Results

### Construction of pBYR2HS-HF-Bet v 1 and Expression of Bet v 1 in *N. benthamiana*

**FIGURE 1:**
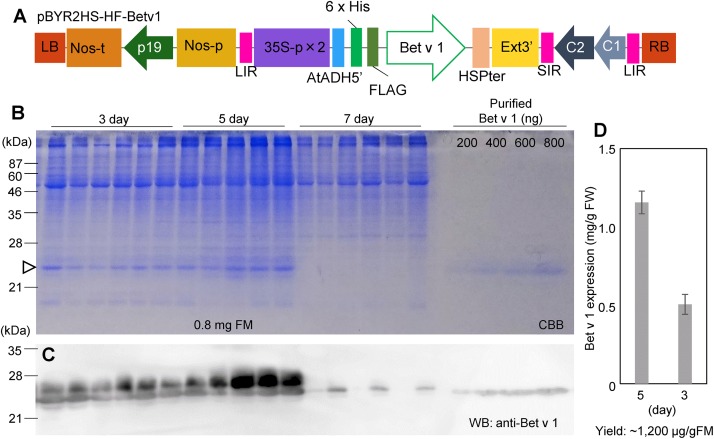
Time-course of Bet v 1 expression in *Nicotiana benthamiana*. The *Nicotiana tabacum* codon-optimized *Bet v 1* gene fused with 6x His and FLAG tag was inserted into pBYR2HS. pBYR2HS-HF-Betv1 was transformed into *Agrobacterium* GV3101. The leaves were harvested at different intervals. Purified Bet v 1 protein expressed in *Brevibacillus brevis* was used to detect the target protein and estimate its concentration. **(A)** Schematic representation of the T-DNA region of the plasmids pBYR2HS-HF-Betv1. 35S-p x 2, CaMV 35S promoter with double-enhanced element; AtADH5′, 5′-untranslated region (UTR) of *Arabidopsis thaliana* alcohol dehydrogenase gene; 6x His, 6x histidine, FLAG, FLAG tag consisting of DYKDDDDK; HSPter, heat shock protein gene terminator; Ext3’, tobacco extensin gene 3′ element; LIR, long intergenic region of bean yellow dwarf virus (BeYDV) genome; SIR, short intergenic region of BeYDV genome; C1/C2, BeYDV ORFs C1 and C2 encoding for replication initiation protein (Rep) and RepA, respectively; LB and RB, the left and right border of the T-DNA region; Nos-p and Nos-t, NOS promoter and terminator; p19, a gene-silencing suppressor gene from tomato bushy stunt virus. **(B)** Total soluble proteins from *N. benthamiana* leaves (0.8 mg FM) were loaded onto an SDS-PAGE gel and electrophoresed. The gel was stained with CBB. An arrowhead indicates the bands corresponding to the Bet v 1 protein. The band corresponding to the large subunit of Rubisco (≈55 kDa) was clearly detected in all samples. **(C)** To confirm that bands indicated by the arrowhead corresponded to the Bet v 1 protein, an anti-Bet v 1 antibody was used. **(D)** The amount of the Bet v 1 protein was calculated based on the band intensity in CBB-stained gels using ImageJ software. Data represent means ± SE (*n* = 5–6).

Leaves of *N. benthamiana* were taken at 3, 5, and 7 days after agroinfiltration with pBYR2HS-HF-Betv1 (Figure 1A). Total soluble proteins prepared from *N. benthamiana* leaves (0.8 mg FM) were detected through the CBB staining (Figure 1B). Total soluble proteins from non-transfected leaves (1.2 mg FM) were also detected through CBB staining (Supplementary Figure S1). Because all soluble proteins were stained by CBB, it may be noticed that, apart from the recombinant target protein (the 25-kDa band), several additional bands were detected on days 3 and 5 post-infiltration. However, no visible band attributable to the target protein was detected on day 7 post-infiltration, probably because several foreign proteins are susceptible to degradation (Doran, 2006).

Immunoblot analysis with an anti-Bet v 1 antibody was carried out to confirm that the 25-kDa band corresponded to the Bet v 1 protein (Figure 1C). Two bands were observed in days 3 and 5 post-infiltration, but only smaller bands were observed in 7-day post-infiltration. The smaller band may be a degraded form of Bet v 1. Bet v 1 expressed in *N. benthamiana* was purified with Ni^+^ column and the purified protein was separated by SDS-PAGE and the gel was stained with CBB (Supplementary Figure S2A). By using this stained gel, estimation of purity was approximately 90%. The band was cut from the gel and the protein in the cut gel was confirmed by LC-MS/MS analysis (Supplementary Figure S2B). A dilution series of the purified recombinant Bet v 1 protein from *B. brevis* was loaded on the same gel (Figure 1B) to estimate the amount of *N. benthamiana-*derived Bet v 1 based on the results obtained with the image analyzer (Figure 1D). Approximately 0.5 and 1.2 mg of Bet v 1/g FM accumulated in *N. benthamiana* leaves agroinfiltrated with pBYR2HS-HF-Betv1 on days 3 and 5 post-infiltration, respectively (Figure 1D). After purification and concentration of Bet v 1, approximately 1.5 mg of Bet v 1 was obtained from 100 g leaves.

**FIGURE 2:**
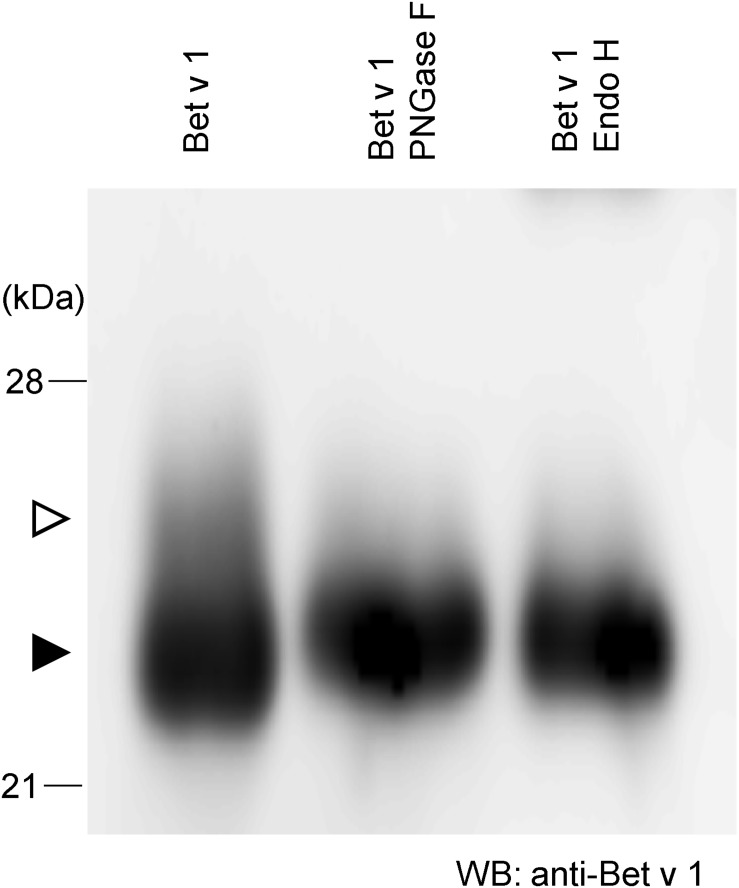
N-glycosylation analysis of Bet v 1 purified from *N. benthamiana*. Purified Bet v 1 was digested in the presence of PNGase F or Endo H and subsequently analyzed by immunoblotting using an anti-Bet v 1 antibody. White and black arrowheads indicate glycosylated and unglycosylated form, respectively.

To achieve more information on the target protein expressed with the Tsukuba system, purified Bet v 1 from *N. benthamiana* was subjected to western blotting. There is a little amount of larger-size Bet v 1 (Figure 2, white arrowhead) whereas majority of Bet v 1 was smaller size (Figure 2, black arrowhead). Bet v 1 contains a single consensus site for *N-*glycosylation (N-S-Y). Then, the protein was treated with either the peptide-*N-*glycosidase F (PNGase F) or the endoglycosidase H (Endo H) and immunoblotted. Endo H hydrolyses the bond connecting the two *N*-acetylglucosamine and cleaves high mannose and hybrid glycans, but not complex glycans. On the other htand, PNGase F cleaves the site of attachment to asparagine of all *N*-linked glycans. After these enzymatic treatments, a single band was observed (Figure 2), suggesting that the small amount of Bet v 1 obtained in *N. benthamiana* was glycosylated with *N-*linked oligosaccharides.

### Construction of pBIC-Betv1 and Expression of Bet v 1 in *B. brevis*

To compare the yield of Bet v 1 recombinant protein production using different protein expression systems and to compare the feature of Bet v 1 derived from plants or from bacteria, the coding sequence of *Bet v 1* was introduced into several pBIC vectors (from pBIC1 to pBIC4). Because expression level of Bet v 1 was 20–100 mg/L in *E. coli*, higher expression system is required (Ferreira et al., 1996). The protein was expressed in *B. brevis* grown in 2SYNm or TMNm culture medium. SDS-PAGE and immunoblot analyses were subsequently performed with anti-Bet v 1 (Supplementary Figure S3A) and anti-polyHis antibodies (Supplementary Figure S3B). Approximately 50 μg of Bet v 1/mL was the yield when *B. brevis* was cultivated in TMNm culture medium (Supplementary Figure S3C).

**FIGURE 3:**
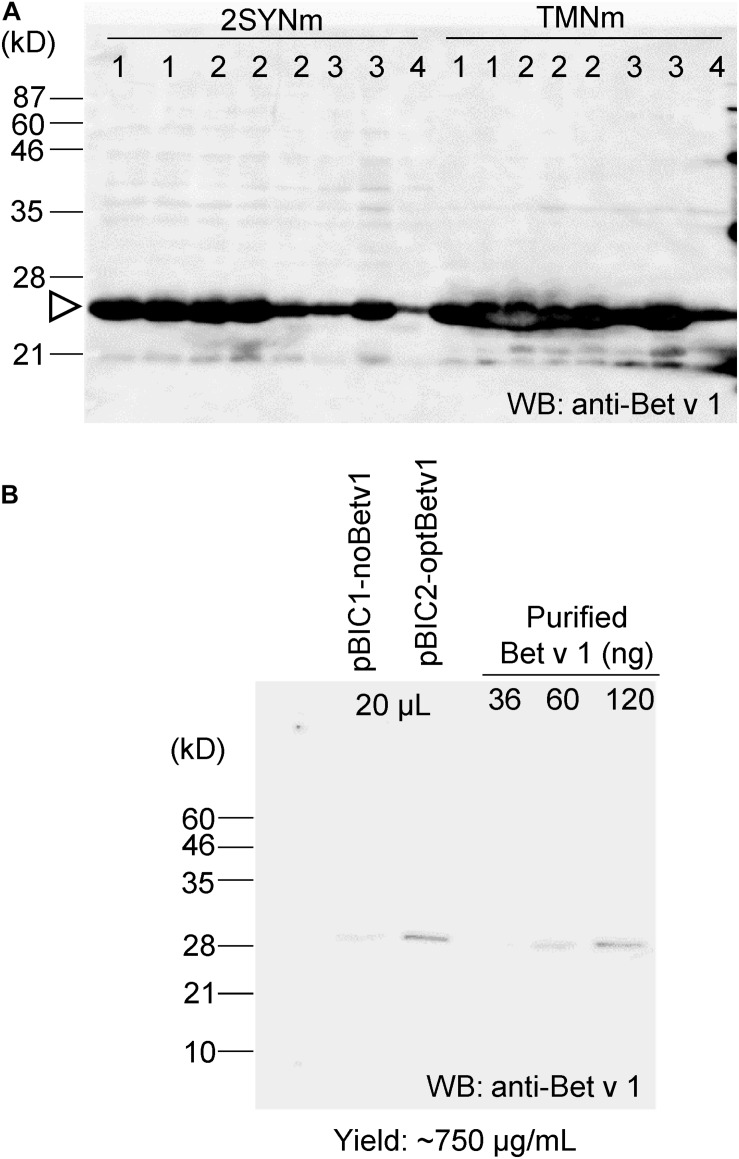
Bet v 1 expression in *B. brevis* was improved by using the *B. brevis* codon-optimized *Bet v 1* gene. The *B. brevis* codon-optimized *Bet v 1* gene was inserted into several pBIC vectors (pBIC1 to pBIC4). **(A)** *B. brevis* harboring each pBIC vector with a codon-optimized *Bet v 1* gene was incubated in 2SYNm or TMNm medium at 33°C for 3 days. After centrifugation, the supernatant was loaded onto SDS-PAGE, and immunoblot analysis using an anti-Bet v 1 antibody was performed. Numbers at the top of the blot indicate the pBIC vector used. **(B)** Protein yield calculation for transformed-pBIC2-optBetv1 in *B. brevis*. *B. brevis* harboring pBIC2-optBetv1 was incubated in TMNm medium. After centrifugation, the supernatant was diluted with the buffer (25-fold dilution), and 20 μL-aliquots of the diluted samples were loaded onto SDS-PAGE. The indicated amounts of purified His-Bet v 1 were also loaded, and immunoblot analysis was performed with an anti-Bet v 1 antibody.

The codon sequence of *Bet v 1* was optimized to increase protein production in *B. brevis*. The *optBet v 1* was introduced into the same pBIC vectors (pBIC1 to pBIC4), and the expression levels of Bet v 1 were then compared (Figure 3A). When pBIC2-optBetv1 was used, very high levels of Bet v 1 production were observed. Thus, the yield of Bet v 1 was further calculated for this system. Total soluble protein samples from pBIC1-noBetv1 (no codon-optimized), pBIC2-optBev1 (*B. brevis* codon-optimized), and a dilution series of purified recombinant Bet v 1 protein from *B. brevis* were loaded onto SDS-PAGE (Figure 3B). Approximately 750 μg of Bet v 1/mL was obtained with *B. brevis* transformed with the pBIC2-optBetv1 vector.

Bet v 1 expressed in *B. brevis* was purified with Ni^+^ column and the purified protein was separated by SDS-PAGE and the gel was stained with CBB (Supplementary Figure S4A). According to this stained gel, estimation of purity was about approximately 70%. The band was cut from the gel and the protein in the cut gel was confirmed by LC-MS/MS analysis (Supplementary Figure S4B). After purification and concentration of Bet v 1, approximately 0.8 mg of Bet v 1 was obtained from 100 mL of culture.

### Reactivity of IgE From Human Sera to Recombinant Bet v 1 From *N. benthamiana* and *B. brevis*

Among 15 serum samples obtained in our previous studies (Yatagai et al., 2013; Kanazawa et al., 2019). 11 samples were positive for specific Bet v 1 IgE, and 4 samples were under the detection limit (<0.1 UA/mL). We excluded from the analysis three samples that were negative for Bet v 1 specific IgE, resulting in 12 serum samples for further analysis.

**FIGURE 4:**
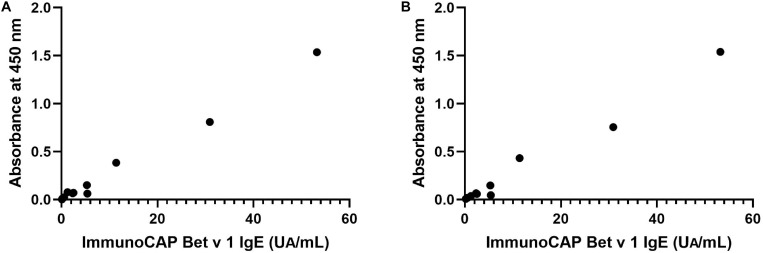
Correlation between specific recombinant Bet v 1 IgE levels determined by ImmunoCAP and OD values in ELISA assays using recombinant allergens derived from *N. benthamiana* **(A)** or *B. brevis* **(B)**. Rho = 0.91, *P* = 0.0001 for *N. benthamiana* and rho = 0.95, *P* < 0.0001 for *B. brevis* in Spearman’s rank correlation analysis.

IgE reactivity to recombinant Bet v 1 from *N. benthamiana* and *B. brevis* was determined by ELISA using 12 serum samples obtained from a general population, and these values were then compared with those measured by ImmunoCAP system, a standard specific IgE detection method (specific IgE test for Bet v 1, Thermo Fisher Diagnostics K.K., Tokyo, Japan). Recombinant Bet v 1 produced in *Escherichia coli* was used to determine specific IgE levels for Bet v 1. Figure 4 shows the correlation between the level of specific Bet v1 IgE determined by ImmunoCAP and the OD values from ELISA assays using recombinant allergens derived from *N. benthamiana* (Figure 4A) and those between ImmnoCAP values and OD values from *B. brevis* (Figure 4B). As it may be noticed, a good agreement between ImmunoCAP and ELISA results using recombinant allergens was observed (Spearman’s rho = 0.91, *P* = 0.0001 for *N. benthamiana* and Spearman’s rho = 0.95, *P* < 0.0001 for *B. brevis* in Spearman’s rank correlation analysis).

## Discussion

In this study, a high amount of the recombinant Bet v 1 protein was obtained by using the Tsukuba system. The recombinant allergen accumulated at a concentration of 1.2 mg/g FM in the leaves of *N. benthamiana*, being this the maximum yield of Bet v 1 reported up to now. IgE reactivity against the recombinant Bet v1 determined by ELISA, which highly correlated with that observed by ImmunoCAP, demonstrated that both plant-produced and *B. brevis-*produced Bet v 1 are recognized by human IgE; therefore, plant-produced Bet v 1 contains IgE epitopes.

Because the birch pollen allergen Bet v 1 is widely known, and much information is available, we selected this protein as a model allergen to confirm the possibility of high protein production levels with the Tsukuba system. Plant-based protein expression systems allow obtaining adequately folded recombinant proteins, being this an additional advantage over *E. coli-*based systems, which were reported to have very low yield (20–100 mg/mL of bacterial culture) (Ferreira et al., 1996). Fewer problems regarding the correct folding and solubility occur when recombinant proteins are expressed in plants. It is reported that refolding steps for production of the anticancer mistletoe lectin viscumin were not required when it is produced in *N. benthamiana* and the steps were required for *E. coli*-derived viscumin (Gengenbach et al., 2019). And it is estimated that the absence of refolding steps saves more than 80% of cost (Gengenbach et al., 2019). Furthermore, the production of plant-expressed recombinant proteins is a cost-effective carbon dioxide re-fixation tool.

Even though recombinant proteins may be obtained using transgenic plants, this is a very time-consuming process, and heterologous protein yield is generally very low (0.01–0.5 mg/g FM). A report demonstrates that transgenic rice expressing in the endosperm the Bet v 1 or the TPC7 were accumulated at 170 and 550 μg/seed, respectively (Ogo et al., 2014). If only seeds were focused, this yield is very high. But about 150 g of shoot FM is required for production of 2 g, about 70 seeds (Takehisa et al., 2004). Thus, about 0.08 and 0.26 mg/g FM of total plants is a yield. On the other hand, almost whole *N. benthamiana* plants can be used, thus, 1.2 mg/g FM is high yield in plants (Figure 1D). Transient expression systems using viral vectors or deconstructed viral vectors are known to increase recombinant protein production (Hefferon, 2017). One deconstructed viral vector system is magnICON, by which ZMapp for *Ebolavirus* infection treatment is produced (Qiu et al., 2014). The Tsukuba system is also a deconstructed viral vector system, harboring a geminiviral replication system with a double terminator (Yamamoto et al., 2018). According to our results, high yield for recombinant Bet v 1 production is obtained in *N. benthamiana*.

*Brevibacillus brevis* is a bacterial host suitable for recombinant protein expression because of its protein secretion ability (Mizukami et al., 2010). Previously, Bet v 1 was expressed in *E. coli* with expression level of 20–100 μg/mL (Ferreira et al., 1996) and this allergen was expressed with the level of 750 μg/mL in *B. brevis* (Figure 3). Based on the observation that IgE antibodies bind to recombinant Bet v 1 derived from both *N. benthamiana* and *B. brevis* (Figure 4), we hypothesize that the molecular conformation of the target protein under our system was conserved. It has been reported that the IgE binding capacity is abolished by alterations of the molecule’s structure (Ferreira et al., 1996). We used several individual sera to cover a wide spectrum of IgE antibodies. Recently, the administration of pharmaceutical-grade tablet formulations of a recombinant Bet v 1 produced by an *E. coli*-expression system was found to be safe and efficacious in the treatment of birch pollen allergic patients (Nony et al., 2015), suggesting that recombinant allergens can replace natural allergen extracts for AIT. There is an advantage of producing recombinant allergens that can be easily produced and unnecessary to reduce the numbers of treatments (Valenta et al., 2016).

When the Bet v 1 protein with the endoplasmic reticulum retention signal (KDEL) was expressed in rice, two bands, corresponding to the glycosylated and the unglycosylated polypeptides, were detected (Ogo et al., 2014). Therefore, it is probable that the upper band found in our SDS-PAGE corresponded to the glycosylated Bet v 1 (Figure 2, white arrowhead). But in this study, no signal was added to Bet v 1. It was described that Bet v 1 contains a single consensus site for N-glycosylation (N-S-Y) (Swoboda et al., 1995). Bet v 1 was mainly localized in the cytoplasm, with a minor portion found in the exine, in the aperture region of pollen grains (El-Ghazaly et al., 1996; Emilson et al., 1996), suggesting that the normal route for Bet v 1 secretion involves the apertures on contact between pollen and the stigmatic surface of the pistil. Thus, only small part of Bet v 1 was glycosylated and most of Bet v 1 may be localized to cytoplasm in this study. These results suggest that glycosylation might occur during allergen secretion.

It is interesting to note that the IgE binding capacity to Bet v 1 derived from *N. benthamiana* and *B. brevis* was similar (Figure 4), even though recombinant Bet v 1 produced in *N. benthamiana* was glycosylated (Figure 2). In some cases, glycan structures are important for the biological activity of allergens. For example, histamine release from passively sensitized basophils of patients was induced by a natural glycosylated allergen of tomato, Lyc e 2, but not by the *E. coli*-derived recombinant protein, suggesting that patients’ IgE recognize carbohydrate determinants (Westphal et al., 2003). Deglycosylated Api g 5, a glycosylated allergen from celery with homology to FAD containing oxidases, was not able to trigger histamine release, but the native allergen did (Bublin et al., 2003). If the glycosylation is the major source of allergen described above, difference of IgE binding capacity to Bet v 1 would be observed. But generally glycosylation is one of the epitopes for IgE. We cannot exclude the possibility of allergen activity of glycosylation even though ImmunoCAP assay showed similar results. The Tsukuba system is one of protein expression system for glycosylated protein.

Bet v 1 biochemical analysis indicates that this allergen belongs to the PR-10 protein family, with a 79 to 83% amino acid sequence identity as compared with other tree allergens such as alder Aln g 1, hornbeam Cor a 1, and hazel Car b 1 (Heath et al., 2015). On the other hand, the major allergens of oak, beech, and chestnut, Que a 1, Fag s 1, and Cas s 1 show a 58, 69, and 75% amino acid sequence similarity with Bet v 1, respectively (Kos et al., 1993; Heath et al., 2015). Birch pollen allergens induce broad and complex patterns of IgE cross-reactivity (Weber, 2004). Because of this cross-reactivity, immunotherapy with Bet v 1 could cover sensitivity to the pollen of several Fagales trees, including hazelnuts, alders, and oaks. A clinical trial to evaluate sublingual immunotherapy to control birch allergy demonstrated significant improvement in medication score during both the birch pollen season and the total tree pollen season, which comprised hazel, alder, and birch seasons (Biedermann et al., 2019). Significant decreases in allergy symptoms of patients with birch allergy were observed by sublingual administration of recombinant Bet v 1 (Nony et al., 2015). By using the Tsukuba system, a high level of recombinant Bet v 1 can be obtained, and given the aforementioned sequence similarities, a high level of protein production for all these Bet v 1 homologous allergens might be achieved.

Many fruits, vegetables, roots, and nuts contain PR-10 proteins. Thus, birch pollen-allergic patients frequently experience hypersensitivity reactions triggered by IgE cross-reacting food sources (Geroldinger-Simic et al., 2011). More than 70% of birch pollen allergic patients reported an allergic reaction to at least 1 of 16 foods analyzed, and among them, apple was the food most commonly related to the induction of allergic symptoms (Geroldinger-Simic et al., 2011). Because the degree of IgE cross-reactivity highly depends on the structural conformation of the allergens, the amino acid similarity, and the epitopes repertoire, some PR-10 proteins including those produced by apple and hazelnut are more often recognized by these patients, as compared to others such as those derived from soybean and celery (Blankestijn et al., 2017). The PR-10 proteins of apple and hazelnut Mal d 1 and Cor a 1 show, respectively, 80 and 59% amino acid sequence similarity with Bet v 1, whereas those of soybean and celery, Gly m 4 and Api g 1, show only 14 and 16%, respectively (Blankestijn et al., 2017). Birch pollen immunotherapy using a mouse model that mimics birch pollen-induced cross-reactivity to Mal d 1 significantly reduced the anaphylaxis induced by Mal d 1 (Utsch et al., 2016). On the other hand, sublingual pollen immunotherapy did not efficiently alter the immune response to pollen-related apple allergen Mal d 1 (Kinaciyan et al., 2007). These data may indicate that a combination of pollen and related food allergens is required for the immunotherapy of individuals with birch pollen allergy associated with food allergy. Given the protein sequence similarity, high levels of food allergens belonging to the PR-10 family might be produced by using our system.

In summary, the Tsukuba system allowed producing the Bet v 1 allergen protein in *N. benthamiana* plants at high levels (1.2 mg/g FM) in a relatively short time (5 days after agroinfiltration), and the purified protein obtained from this plant species displayed identical immunological properties against human IgE as compared with recombinant Bet v 1 protein produced in *B. brevis*.

## Data Availability Statement

All datasets generated for this study are included in the article/Supplementary Material.

## Ethics Statement

The studies involving human participants were reviewed and approved by University of Tsukuba and University of Fukui. The patients/participants provided their written informed consent to participate in this study.

## Author Contributions

YY, MK, AY, TN, and HY performed the experiments and analyzed the data. YK, HM, NH, and SF prepared human serum. EN and KM participated in study design and coordination, and writing of the manuscript. All authors have approved the final version of the manuscript.

## Conflict of Interest

The authors declare that the research was conducted in the absence of any commercial or financial relationships that could be construed as a potential conflict of interest.

## Abbreviations

AIT: allergen immunotherapy
CBB: Coomassie brilliant blue
Endo H: endoglycosidase H
FM: fresh mass
IgE: immunoglobulin E
OD: optical density
PNGase F: peptide-*N-*glycosidase F
PR-10: pathogen-related protein 10
TMV: tobamovirus
TPC: tree pollen chimera.
