# Supplementary material for: High-Yield Production of the Major Birch Pollen Allergen Bet v 1 With Allergen Immunogenicity in Nicotiana benthamiana
Source: Front Plant Sci. 2020 Apr 2;11:344. doi: 10.3389/fpls.2020.00344 (PMC7142267; doi:10.3389/fpls.2020.00344)
Supplement: Supplementary file 1 [file Data_Sheet_1.PDF]

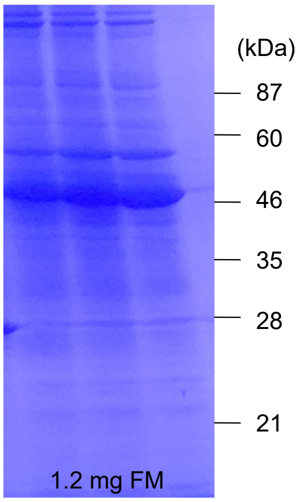

Supplemental Figure S1. Coomassie Brilliant Blue (CBB) staining of total soluble proteins from non-transfected *N. benthamiana*. Total soluble proteins were prepared from non-transfected plants and separated in SDS-PAGE. The gel was stained by CBB.

A

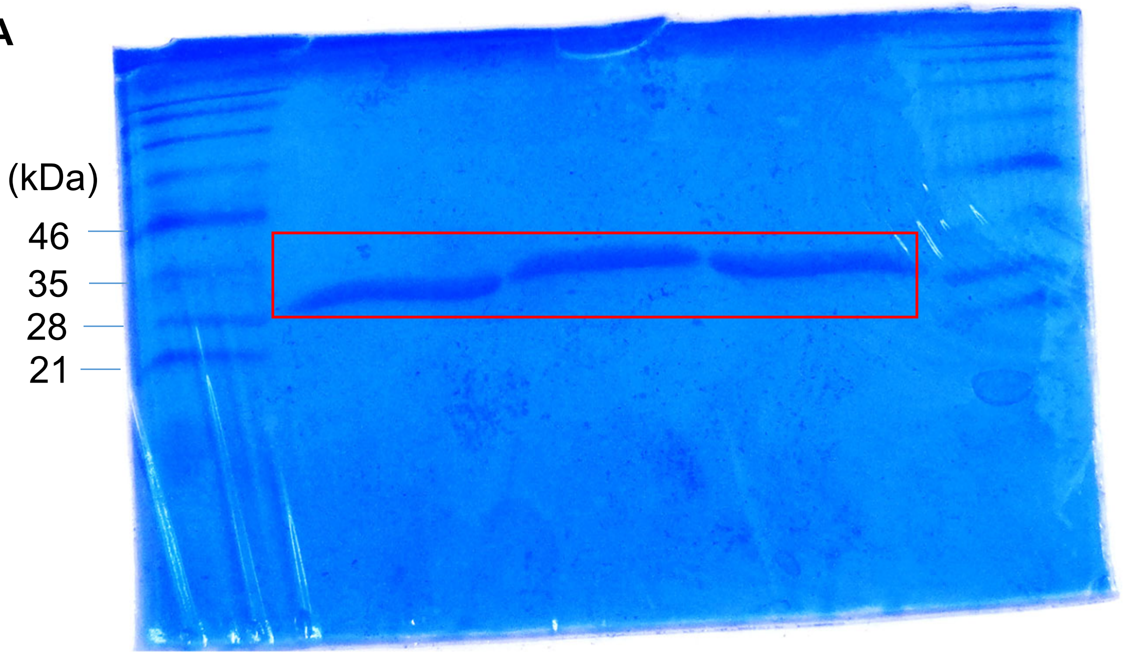

B

MATRIX SCIENCE MASCOT Search Results

Protein View: P15494.2

RecName: Full=Major pollen allergen Bet v 1-A; AltName: Full=Allergen Bet v I-A; AltName: Allergen=Bet v 1-A

**Database:**	NCBIprot
**Score:**	468
**Monoisotopic mass (M_r):**	17560
**Calculated pI:**	5.39
**Taxonomy:**	Betula pendula

This protein sequence matches the following other entries:

- CAA33887.1 from Betula pendula
- CAB02153.1 from Betula pendula
- CAB02154.1 from Betula pendula
- CAA07321.1 from Betula pendula

Sequence similarity is available as an NCBI BLAST search of P15494.2 against nr.

Search parameters

**MS data file:**	masBF.tmp
**Enzyme:**	Trypsin: cuts C-term side of KR unless next residue is P.
**Variable modifications:**	Carbamidomethyl (C), Oxidation (M)

Protein sequence coverage: 64%

Matched peptides shown in ***bold red***.

```
  1 MGVFNYETET TSVIPAARLF KAFILDGDNL FPKVAPQAIS SVENIEGNGG
 51 PGTIKKISFP EGFPFKYVKD RVDEVDHTNF KYNYSVIEGG PIGDTLEKIS
101 NEIKIVATPD GGSILKISNK YHTKGDHEVK AEQVKASKEM GETLLRAVES
151 YLLAHSDAYN
```

Supplemental Figure S2. Confirmation of Bet v 1 purified from *Nicotiana benthamiana* by LC-MS/MS analysis. (A) Bet v 1 was expressed in *N. benthamiana* and purified with Ni^{+} column. After staining with CBB, the band surrounded by red box was excised for LC-MS/MS analysis. (B) MASCOT search results by using the data of LC-MS/MS analysis. Bet v 1-A was top score with coverage of 64%.

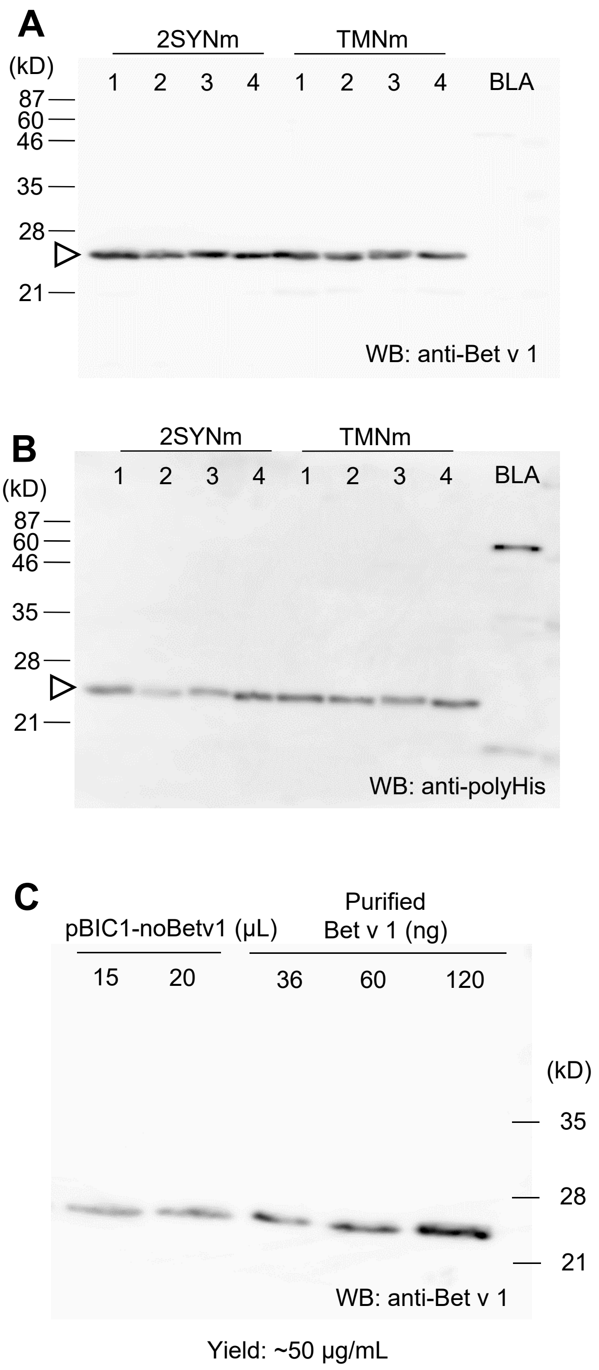

Supplemental Figure S3. Expression of Bet v 1 in *Brevibacillus*. A natural *Bet v 1* gene was inserted into pBIC1 to 4. The number at the top of the blot indicates each pBIC vector. *Brevibacillus* harboring each pBIC vector with a natural *Bet v 1* gene was incubated in 2SYNm or TMNm medium at 33 ºC for 3 days. After centrifugation, supernatant was loaded onto SDS-PAGE and immunoblot analysis was performed. The Bet v 1 protein was detected by anti-Bet v 1 antibody (A) or anti-polyHis antibody (B). BLA, *Brevibacillus licheniformis* α-amylase, was expressed as a positive control. (C) Comparison of expression of Bet v 1 with purified Bet v 1. *Brevibacillus* harboring pBIC1-noBetv1 was incubated in TMNm medium. After centrifugation, the media was diluted with the buffer at 25 fold. Then, 15 or 20 µL of the diluted media was loaded onto SDS-PAGE. And, the indicated amount of purified His-Bet v 1 was also loaded onto the same SDS-PAGE and immunoblot analysis was performed with anti-Bet v 1 antibody.

A

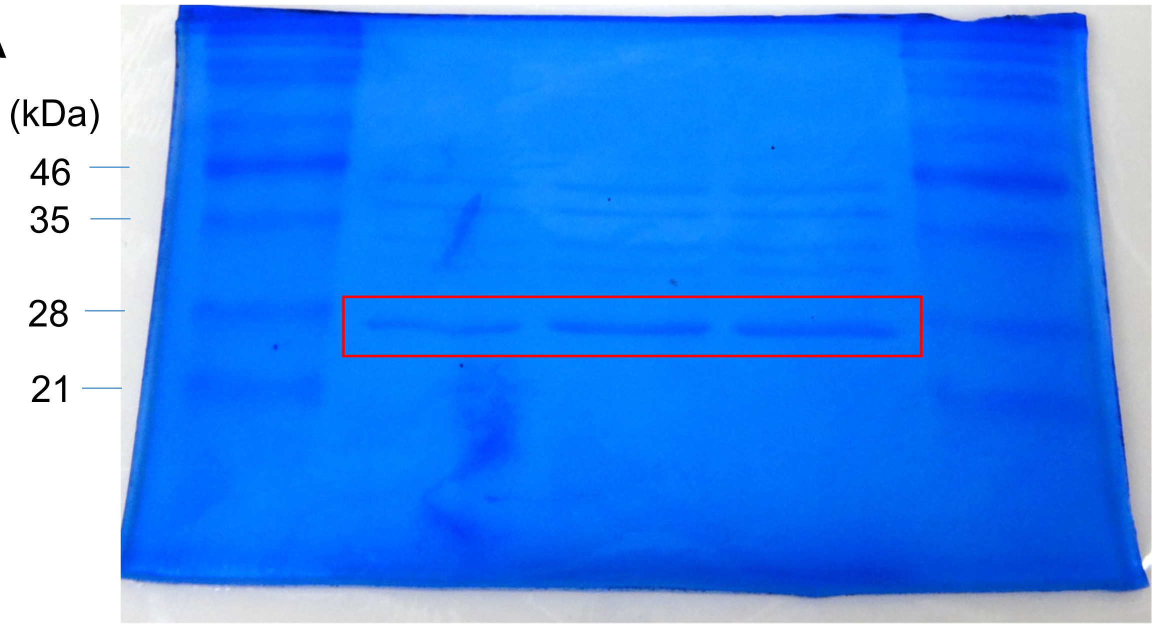

B

MASCOT Search Results

Protein View: gi|114922

RecName: Full=Major pollen allergen Bet v 1-A; AltName: Full=Allergen Bet v I-A; AltName: Allergen=Bet v 1-A

**Database:**	NCBInr
**Score:**	406
**Nominal mass (M_r):**	17560
**Calculated pI:**	5.39
**Taxonomy:**	Betula pendula

This protein sequence matches the following other entries:

- gi|17938 from Betula pendula
- gi|1542857 from Betula pendula
- gi|1542859 from Betula pendula
- gi|4006949 from Betula pendula

Sequence similarity is available as an NCBI BLAST search of gi|114922 against nr.

Search parameters

**Enzyme:**	Trypsin: cuts C-term side of KR unless next residue is P.
**Variable modifications:**	Carbamidomethyl (C), Oxidation (M)

Protein sequence coverage: 65%

Matched peptides shown in ***bold red***.

```
  1 MGVFNYETET TSVIPAARLF KAFILDGDNL FPKVAPQAIS SVENIEGNGG
 51 PGTIKKISFP EGFPFKYVKD RVDEVDHTNF KYNYSVIEGG PIGDTLEKIS
101 NEIKIVATPD GGSILKISNK YHTKGDHEVK AEQVKASKEM GETLLRAVES
151 YLLAHSDAYN
```

Supplemental Figure S4. Confirmation of Bet v 1 purified from *Brevibacillus brevis* by LC-MS/MS analysis. (A) Bet v 1 was expressed in *B. brevis* and purified with Ni^{+} column. After staining with CBB, the band surrounded by red box was excised for LC-MS/MS analysis. (B) MASCOT search results by using the data of LC-MS/MS analysis. Bet v 1-A was top score with coverage of 65%.

Table S1. Primers used for plasmid construction.

Name of primer	DNA sequence (5'-to-3')
pBIC-HIS-Betv1wt-F	GATGACGATGACAAAATGGGTGTTTTCAATTACGAAACTGAGGC
pBIC-HIS-Betv1wt-R	CATCCTGTTAAGCTTTTAGTTGTAGGCATCGGAGTGTGACAAAA
pBIC-HIS-Betv1ABb-F	GATGACGATGACAAAATGGGCGTCTTCAACTACGAGACGGAG
pBIC-HIS-Betv1ABb-R	CATCCTGTTAAGCTTTTAGTTGTACGCATCGCTATGCGCCAGC
pBYR2HS-His24	ACTGTTGATAGTCGATGCATCATC
pRI201-Betv1ANt-R	ATTCAGAATTGTCGACTTAATTGTATGCATCAGAGTGAGCGAGA
